# Mucoperiosteal Flap Healing During Vertical Bone Augmentation using Titanium Mesh: A Study in Beagle Dogs

**DOI:** 10.1016/j.identj.2025.04.004

**Published:** 2025-05-09

**Authors:** Lan Wang, Yang Zhou, Renshengjie Zhao, Keming Xiao, Maoyun Zeng, Xinyu Xie, Qiqi Liu, Ke Yu

**Affiliations:** The Affiliated Stomatological Hospital, Southwest Medical University, Luzhou, China

**Keywords:** Wound healing, Wound dehiscence, Keratinized gingiva, Guided bone regeneration, Titanium membrane, Periosteal releasing incision

## Abstract

**Introduction and aims:**

The periosteal releasing incision (PRI) is often used in guided bone regeneration (GBR) with titanium (Ti) mesh to reduce the tension of mucoperiosteal flaps (MPF) and close the wound, but it can easily lead to keratinized gingiva (KG) narrowing and a high wound dehiscence rate. Therefore, it is necessary to explore alternative methods such as open healing. The aim of this study was to observe whether a MPF without a PRI and primary closure could heal on a titanium membrane surface in vertical bone augmentation and to measure the change in KG width.

**Methods:**

The bilateral mandibular second, third, and fourth premolars and first molars were extracted from 6 beagle dogs. After 3 months, 4 sites were prepared on each side of the mandible to perform vertical bone augmentation and divided into 4 groups using a randomized block design. Group A: Bio-oss + Ti-membrane + Bio-gide + PRI and closing MPF; Group B: Bio-oss + Ti-membrane + Bio-oss + Bio-gide + PRI and closing MPF; Group C: Bio-oss + Ti-membrane + Bio-oss + Bio-gide + no PRI and unclosing MPF; and Group D: Ti-membrane + Bio-gide + no PRI and closing MPF. The study parameters were wound healing rate and dehiscence rate, KG widths, histologic analysis of gingiva, and analysis of the Ti-membranes surface.

**Results:**

The wound dehiscence rates in groups A, B, and D were 50%, 41.67%, and 8.3%, respectively, and there was no statistical difference between group A and group B (*P* = 1.000). And in group C, only 1 wound exhibited incomplete soft-tissue closure of the gum (8.3%). The KG width in group C increased by 1.77 ± 0.37 mm, while the KG width in group B decreased by 4.37 ± 0.45 mm, and there was a significantly statistical difference between the 2 groups (*P* < .001). Compared to group B, the new gingiva in group C had better histological performance.

**Conclusion:**

An MPF without PRI and primary closure can heal on the clean surface of the Ti membrane during vertical bone augmentation, and the KG can widen.

**Clinical Relevance:**

The results have implications for the postoperative wound management of GBR supported by a titanium membrane.

## Introduction

Bone augmentation techniques, such as guided bone regeneration (GBR), autogenous block bone grafting, distraction osteogenesis, and the alveolar ridge split technique, have solved the problem of bone deficiency in dental implant treatment.^1^ However, owing to the difficulties of these techniques and the preference of surgeons, GBR is currently the most widely used bone augmentation technique.^2^ The GBR technique only using resorbable barrier membranes and granular bone can achieves good results for small bone defects.^3^ The commonly used xenogeneic granular bone in the dental field is deproteinized bovine bone which is commercially available as Bio-oss (Geistlich, Switzerland). Its porous structure possesses excellent mechanical properties and promotes bone healing through osteoconduction. Meanwhile, the porous structure has a huge surface area, which is conducive to the growth of new blood vessels.^4^ Collagen membrane is the most frequently used resorbable barrier membrane with good bioactivity, but its low mechanical strength makes it difficult to maintain space for an appropriate length of time.^5^ Another major drawback of collagen membrane is the rate of degradation, leading to an early loss of barrier function.^6^ Therefore, for large-scale and vertical bone defects, relying solely on granular bone makes it difficult to avoid membrane collapse and displacement of the granular bone.^7^ Accordingly, various types of titanium meshes or titanium membranes, which are strong support devices, are used to solve the problem of spatial collapse in GBR.^8^, ^9^ Titanium mesh/membrane exhibits favorable mechanical properties, and its high stiffness and strength enable space support for osteogenesis. Its stability is essential to sustain bone graft volume during wound healing.^10^ Furthermore, titanium’s corrosion resistance and low cytotoxicity enhance its compatibility with tissues.^11^, ^12^ However, wound dehiscence and exposure of the titanium mesh are a problem with reports of titanium mesh exposure rates as high as 51%.^13^ Certainly, the recently developed biodegradable GBR membranes made of pure magnesium may be an alternative.^14^

Many factors are associated with titanium mesh exposure, such as wound soft tissue tearing, infection, sharp mechanical damage, and the irregular shape, surface biocompatibility, thickness, and deformation of the titanium mesh, all of which are related to the primary closure of the wound.^15^, ^16^ At present, the most commonly used method for reducing the tension of the mucoperiosteal flap to close the wound is the periosteal releasing incision, which involves cutting off the periosteum and submucosa to relieve flap tension.^17^ However, this type of incision can lead to postoperative complications in the soft tissue, such as edema and hematoma, causing discomfort to patients. Another disadvantage of this incision is that it interrupts blood supply from the periosteal layer to the gingival flap, particularly when the periosteal release incision penetrates deep into the submucosal layer and even the muscular layer.^17^ Xiong et al. proposed that angiogenesis played an important role in diabetic wound healing.^18^ Yang et al. proposed that more vascular endothelial cell markers (CD31) could be observed in wounds with better healing.^19^ Therefore, although the mucoperiosteal flap has fully reduced tension and achieved primary closure of the wound, some patients still experience exposure of the titanium mesh, which is related to insufficient blood supply to the gingival flap, leading to poor healing or even necrosis of the gingiva. The final drawback of the periosteal releasing incision is the narrowing of the keratinized gingiva and shallowing of the vestibular sulcus due to the coronal movement of the mucoperiosteal flap.^20^

Considering the aforementioned drawbacks of the periosteal releasing incision, we wondered if it was possible to avoid using this incision in a GBR with titanium mesh or titanium membrane to reduce the tension of the mucoperiosteal flap. Admittedly, in this case, primary wound closure may not be achieved and the wound will be open. However, clinical studies have confirmed that gums can heal on the surface of titanium mesh.^21^, ^22^ Therefore, this study aimed to explore whether the wound could heal and whether the gingiva could widen on the surface of the titanium membrane in vertical augmentation without a periosteal releasing incision and primary closure of the gingival flap.

## Methods

### Animals

This study was approved by the Animal Ethics Committee of Southwest Medical University, Luzhou, China (No. SWMU20230049). The study followed the ARRIVE (Animal Research: Reporting of In Vivo Experiments) guidelines.^23^

Six healthy male beagle dogs (12 ± 2 kg) were uniformly fed regular pellet feed and drinking water obtained from Changzhou Beile Experimental Animal Breeding Co. Ltd, China (licence: SYXK(S)2013-0065). For tooth extraction and bone augmentation, the dogs were subjected to general anesthesia with pentobarbital sodium (intramuscular [IM], 30 mg/kg; Sigma, USA) and received antibiotics (IM, 40,000 unit/kg, penicillin potassium; Macklin, Shanghai, China) for 7 days after each treatment.

### Sample size calculation

The sample size of this study was calculated using the formula, n = 2 (t*_α_* + t_1-_*_β_*)^2^ *σ*^2^/*δ*^2^. To compare the changes in the width of KM at different sites, the required sample size was determined based on the mean difference of 1.2 mm and SD 1.03 mm with α of 0.05, power (*β*) of 0.8.^24^ The t represents the critical value from the t-distribution. In initial calculations, it is commonly assumed that the t-distribution approximates the normal distribution. Therefore, in the present calculation, t_α_ ≈ 1.96 and t_1−β_ ≈ 0.84. The sample size of each group was calculated to be twelve, and 6 beagle dogs were used in the study based on the experimental design.

### Surgical procedures

All surgical procedures were performed by an experienced implant surgeon. The second premolar (P2), third premolar (P3), fourth premolar (P4), and first molar (M1) on each side of the mandible were extracted from beagle dogs. After 3 months of bone and gingival healing, vertical bone augmentation with GBR using a titanium membrane support was performed. Four sites were prepared on each side of the mandible, with a width of 8 mm for each site, spacing of more than 4 mm between sites, and spacing of more than 2 mm between the sites and the natural teeth. Using the randomized block design method, the 4 sites on each side of the mandible were allocated into 4 groups (Supplementary table 1): Group A: Bio-oss + Ti-membrane + Bio-gide + PRI and closing MPF, Group B: Bio-oss + Ti-membrane + Bio-oss + Bio-gide + PRI and closing MPF, Group C: Bio-oss + Ti-membrane + Bio-oss + Bio-gide + no PRI and unclosing MPF, and Group D: Ti-membrane + Bio-gide + no PRI and closing MPF.

**Fig. 1:**
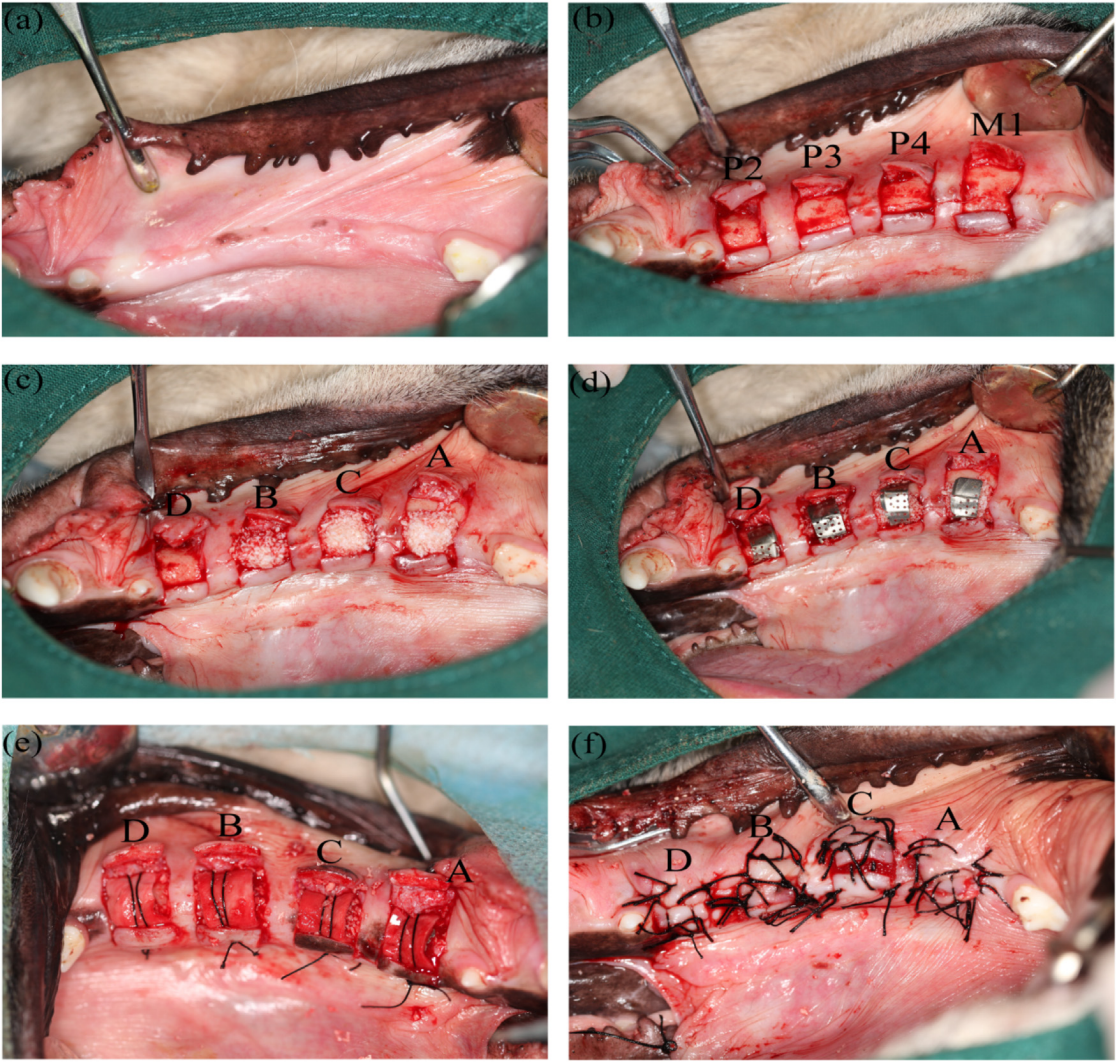
Surgical procedure of the experiments. Group A: Bio-oss + Ti-membrane + Bio-gide + PRI and closing MPF; Group B: Bio-oss + Ti-membrane + Bio-oss + Bio-gide + PRI and closing MPF; Group C: Bio-oss + Ti-membrane + Bio-oss + Bio-gide + no PRI and unclosing MPF; and Group D: Ti-membrane + Bio-gide + no PRI and closing MPF. (A) The healing gingiva and wounds; (B) H-shaped incisions were made at the P2, P3, P4, and M1 sites and the mucoperiosteal flaps were raised; (C) Granular bone was piled in groups A, B, and C; (D) Titanium membranes covered in groups A, B, C, and D; (E) Collagen membrane covered and was fixed in groups A, B, C, and D; (F) The wounds were sutured and closed in different ways.

At each site, a horizontal incision was made at the crest of the alveolar ridge, and 2 vertical incisions approximately 15 mm in length were made on the mesial and distal sides of the site. The mucoperiosteal flaps were raised on the buccal and lingual sides (Figure 1A and 1B). In groups A, B, and C, granular bone (Bio-oss, Geistlich, Switzerland) was piled 3 mm high on the surface of the alveolar bone (Figure 1C), then a porous titanium membrane (CTi-mem, Neobiotech, Korea), 7 mm in length and 5 mm in width, covered the surface of the granular bone; however, in group D, the same titanium membrane covered the surface of the alveolar bone directly (Figure 1D). In groups B and C, an additional 1 mm thick granular bone covered the surface of the titanium membrane. In the 4 groups, a collagen membrane (Bio-gide, Geistlich, Switzerland) covered and was fixed through a horizontal mattress suture with a 4-0 silk thread (Figure 1E). In groups A and B, periosteal-releasing incisions were made on the buccal mucoperiosteal flaps, and the wounds were closed tightly with vertical mattress sutures alternated with interrupted sutures (Figure 1F). In group C, owing to the lack of a periosteal releasing incision, the wound was not closed with a 3 mm wide fissure, a 2 mm thick gelatin sponge (Fukangsen, Guilin, China) was used to cover the fissure, and the buccal and lingual flaps were fixed with an 8-character suture and interrupted sutures (Figure 1F). In group D, the incision was closed with interrupted sutures without a periosteal releasing incision (Figure 1F).

### Clinical observation and KG width measurement

After surgery, the beagle dogs were kept warm in separate cages placed in an environment of 25 centigrade and given soft food to eat. And beagle dogs were given an intramuscular injection of penicillin (40,000 unit/kg; Macklin, Shanghai, China) daily for 7 days after surgery. The wounds were alternately flushed with a mouthwash containing 1.2 mg/mL chlorhexidine, 0.2 mg/mL metronidazole and normal saline 3 times a day for 3 weeks. The appearance, behavior, reactivity, and social interactions of the dogs were observed throughout the experimental period. Wound dehiscence was observed in groups A, B, and D, while wound healing was observed in group C.

**Fig. 2:**
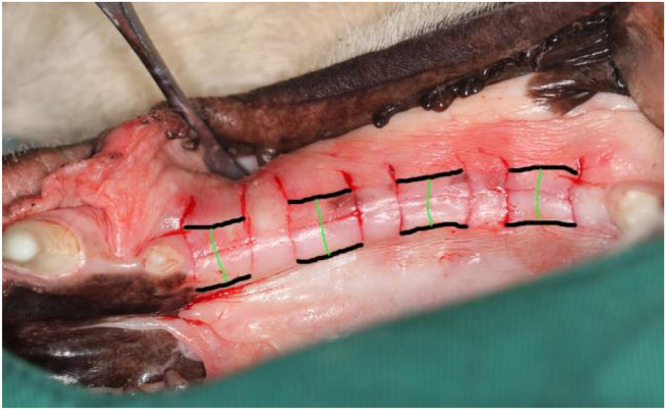
Keratinized gingiva width measurement. The black lines represent the buccal and lingual mucogingival junctions, and the green lines represent the arc length of the keratinized gingiva at the midline of the experimental sites.

The midline of each site in the mesial-distal direction was selected as the location for measuring the width of the keratinized gingiva. The gingiva was tightly covered with a silk thread at the measurement point and cut from the buccal mucogingival junction to the lingual mucogingival junction. A Vernier caliper was used to measure the length of the silk thread as the width of the keratinized gingiva. The measurements were performed 3 times by the same experimenter, and the average value was calculated (Figure 2).

### Gingiva sample preparation and section observation

**Fig. 3:**
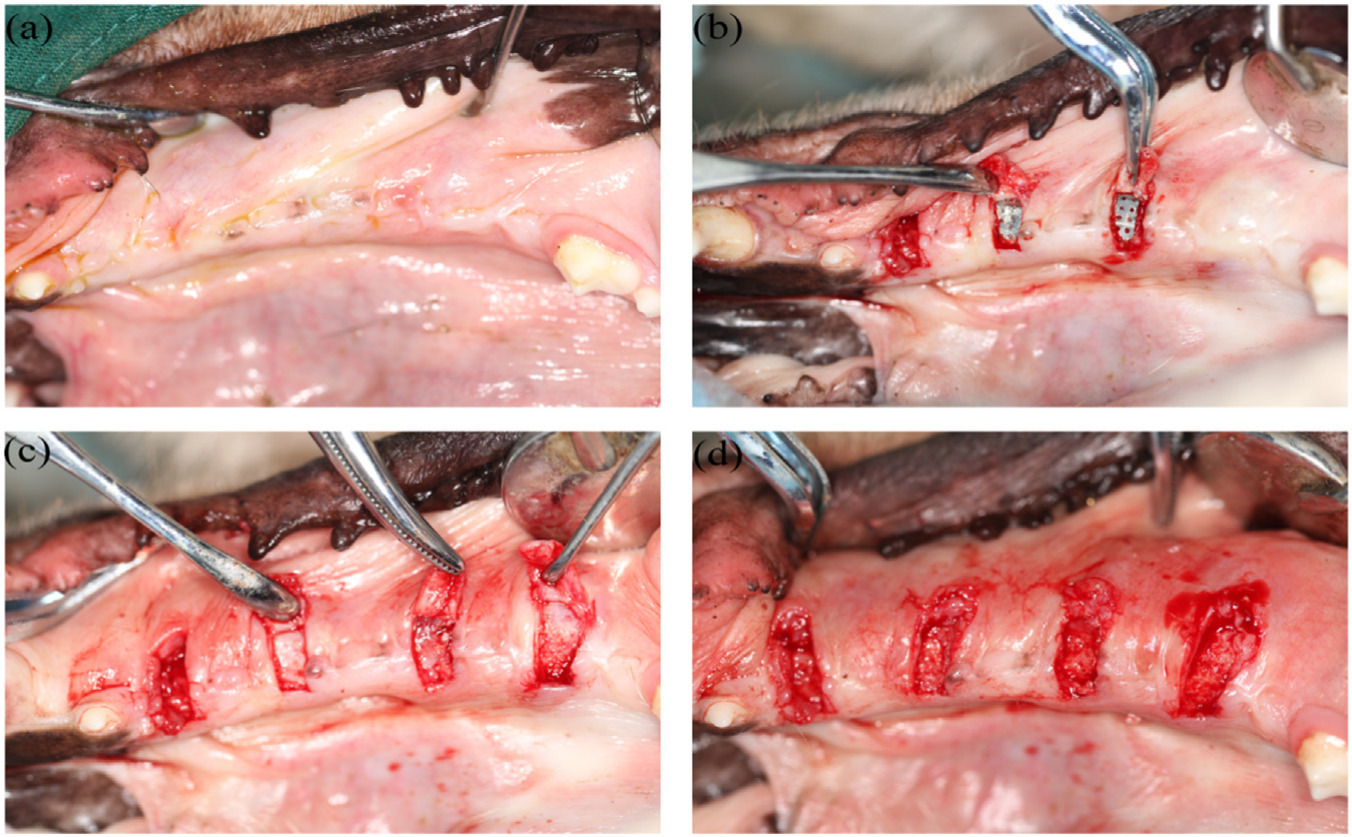
Gingiva sample preparation. (A) The wounds healed 1 month after surgery; (B) The gingiva was cut open to expose the titanium membrane; (C) The titanium membrane was removed and fiber wrapped the granular bone with a hard texture; (D) The gingiva was cut off.

One month after surgery, the 6 dogs were anesthetized and the gingiva (approximately 3 mm wide, 6 mm long) on the titanium membrane was cut off at the sites where the wounds had completely healed (Figure 3). Normal gingiva of the same size was obtained from the area between the 2 experimental sites as a control. All the samples were fixed in 4% paraformaldehyde, embedded in paraffin, and sectioned into 4 μm thick slices.

*Hematoxylin and eosin (HE) staining*. The slices were soaked in xylene Ⅰ, Ⅱ, and Ⅲ for 10 min each for dewaxing. The slices were then sequentially immersed in 100%, 95%, 90%, 80%, and 75% ethanol for 5 min each to achieve sufficient hydration. Next, the slices were stained with hematoxylin dye and eosin dye for 5 minutes each. Subsequently, gradient dehydration was performed on the slices using 75%, 85%, 95%, and 100% ethanol. Finally, the slices were soaked in xylene, air dried, sealed with neutral gum, and observed under an optical microscope.

*Masson staining*. The slices were soaked in xylene Ⅰ, Ⅱ, and Ⅲ for 10 min each for dewaxing. The slices were then sequentially immersed in 100%, 95%, 90%, 80%, and 75% ethanol for 5 min each to achieve sufficient hydration. Next, the slices were steeped in hematoxylin and Ponceau magenta staining solutions for 2 and 5 min, respectively. The slices were rinsed with phosphomolybdic acid solution and a weak acid working solution for 1 min, then stained with 1% toluidine blue staining solution for 5 min. Subsequently, the slices were dehydrated in 95% and 100% ethanol. Finally, the slices were soaked in xylene, air dried, sealed with neutral gum, and observed under an optical microscope.

*Immunofluorescence*. The slices were sequentially dewaxed and dehydrated in xylene and ethanol. The samples were permeabilized with 0.3% Triton X-100. Slices were then blocked with 5% bovine serum albumin (BSA) at room temperature for 1 h. The slides were incubated with primary antibodies against CD31 (Bioss, Cat# bs-0195R, RRID: AB_10856841) at 4°C overnight. Subsequently, the slides were washed 3 times with phosphate-buffered saline (PBS) for 5 min each before staining with secondary antibodies for 2 h. The slides were washed 3 times with PBS for 5 minutes each. Next, DAPI staining solution was applied to stain the nuclei of the tissue. Finally, the slides were sealed with an anti-fade solution. The slices were observed under a confocal microscope.

### Scanning electron microscopy (SEM) observation

Titanium membranes were selected from healed and non-healed wounds in group C for SEM. The titanium membranes were fixed in 3% glutaraldehyde for 2 days. The surfaces of the titanium membranes that were in contact with the gingiva were observed using SEM.

### Statistical analysis

SPSS (version 27.0, Chicago, Illinois, USA) was used for statistical analysis. The Shapiro-Wilk test was used to test the normality of the data. Normally distributed data are described as mean and standard deviation (SD) and are analyzed using analysis of variance (ANOVA) or a parametric t-test. Non-normally distributed data are described as median with interquartile range (IQR) and are analyzed using the nonparametric Mann-Whitney U test. Categorical variables are expressed as rates (percentages). The chi-square test/Fisher’ s precision probability test was used to determine whether there was a difference between the samples. Differences were considered statistically significant at *P* < .05.

## Results

### Clinical observation

**Fig. 4:**
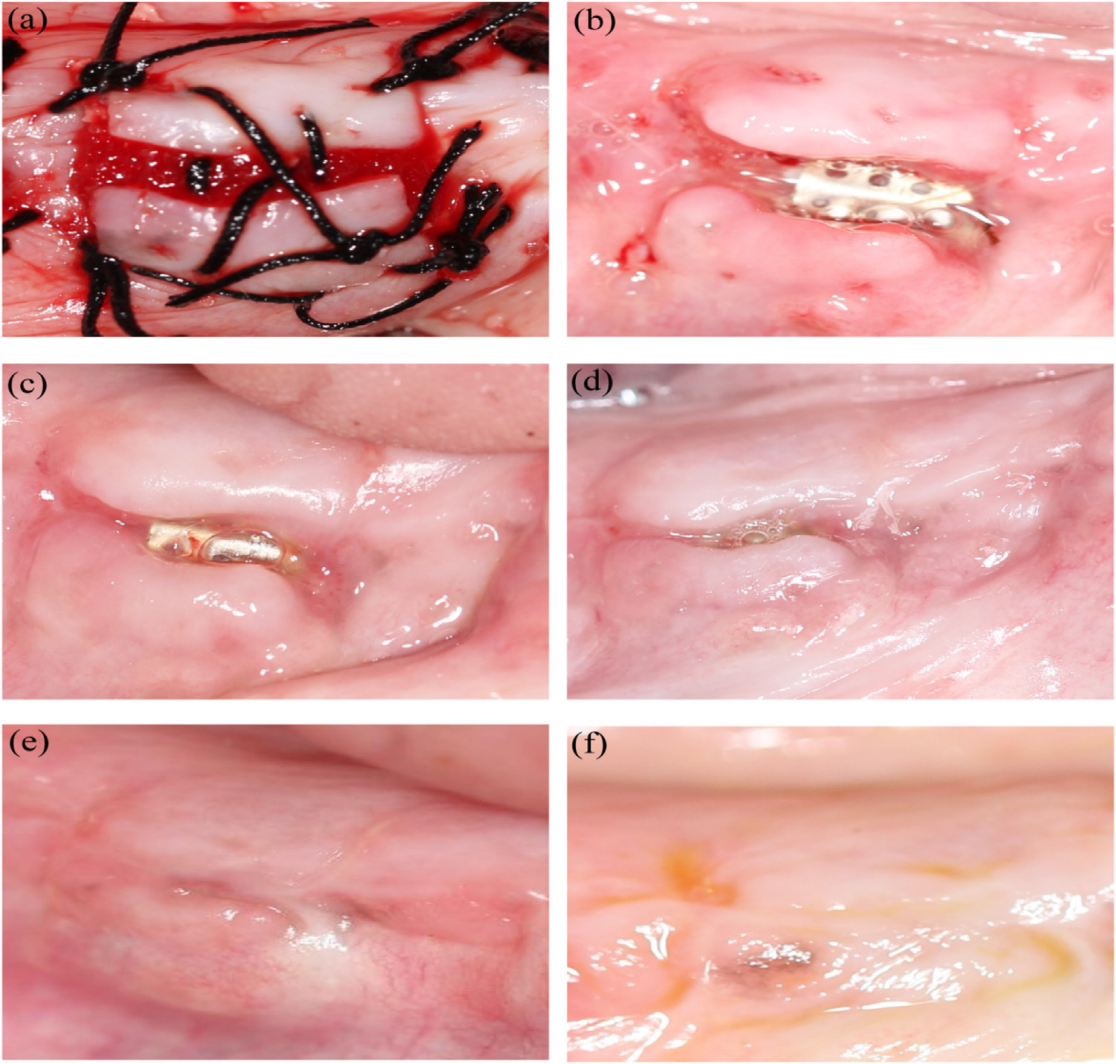
The wound healing process in the experimental site without primary closure of the mucoperiosteal flap: (A) immediately, (B) 7 days, (C) 12 days, (D) 18 days, (E) 21 days, and (F) 1 month after surgery.

**Fig. 5:**
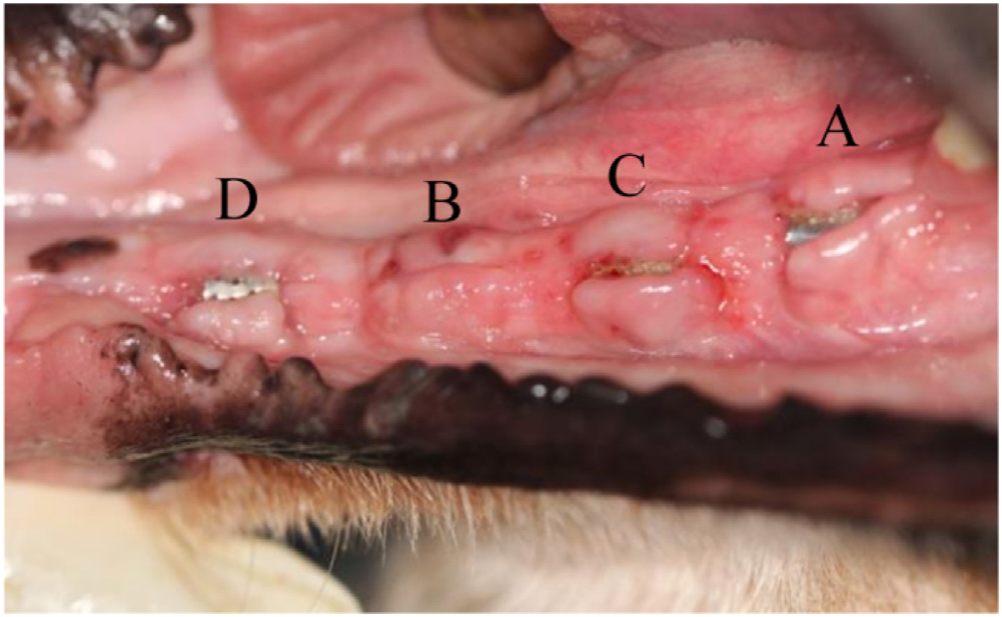
Non-healing wound in group C and wound dehiscence in groups A and D 1 month after surgery.

Throughout the experiment, all dogs were in good physical condition and did not develop acute systemic infections. In group C, the gelatin sponge covering the surface of the wound fell off within 3 days, the collagen membranes were absorbed within 1 week, and all titanium membranes were exposed 7 days postoperatively (Figure 4A and 4B). The fissures gradually narrowed (Figure 4C, 4D, and 4E) and finally fully healed approximately 1 month after surgery (Figure 4F). Only 1 wound at the P3 site in group C exhibited incomplete soft-tissue closure of the gum and remained in a state of titanium membrane exposure (Figure 5), accounting for 8.3%.

In groups A, B, and D, the wounds began to swell and gradually subsided after 1 week, and fully healed after 2 weeks. Only 1 site, P2 in group D, showed wound dehiscence, accounting for 8.3%. The wound dehiscence rates in groups A and B were 50.0% and 41.7%, respectively, with dehiscence occurring at each tooth position (Table 1; Figure 5), and there was no statistical difference between the 2 groups (*P* > .05).

**Table 1:** The outcomes of all wounds and changes in the width of keratinized gingiva of completely healed wounds.

Group	A (N = 12)	B (N = 12)	C (N = 12)	D (N = 12)
Wound healing	6 (50.0%)	7 (58.3%)	11 (91.7%)	11 (91.7%)
Wound dehiscence	6 (50.0%)	5 (41.7%)*	-	1 (8.3%)
Incomplete soft-tissue closure	-	-	1 (8.3%)	-
Keratinized gingiva width (mm)	N = 6	N = 7	N = 11	N = 11
T0	9.09 ± 0.60	8.92 ± 0.79	8.30 ± 0.51	8.55 ± 0.49
T1	4.36 ± 0.64	4.55 ± 0.81	10.06 ± 0.68	9.11 ± 0.52
T1 − T0	−4.74 ± 0.45	−4.37 ± 0.45†	1.77 ± 0.37	0.56 ± 0.39

N, sample size; T0, before GBR surgery; T1, 1 month after GBR surgery.

⁎ Compared with group A, the Chi-Square Test, *P* = 1.000.

† Compared with group C, parametric t-test, *P* < .001. The number of completely healed wounds at each tooth position for each group: Group A: n (P2)=1, n (P3)=2, n (P4)=1, n (M1)=2; Group B: n (P2)=1, n (P3)=2, n (P4)=2, n (M1)=2; Group C: n (P2)=3, n (P3)=3, n (P4)=2, n (M1)=3; Group D: n (P2)=2, n (P3)=3, n (P4)=3, n (M1)=3.

In the 3 vertical bone augmentation groups (groups A, B, and C), after removing the titanium membranes, the fibers wrapped the granular bone with a hard texture (Figure 3C).

### Keratinized gingiva width measurement

The width of the keratinized gingiva decreased in both groups A and B, where periosteal releasing incisions were employed. The width of the keratinized gingiva decreased by 4.37 ± 0.45 mm in group B and increased by 1.77 ± 0.37 mm in group C (*P* < .001; Table 1).

### Histological evaluation

**Fig. 6:**
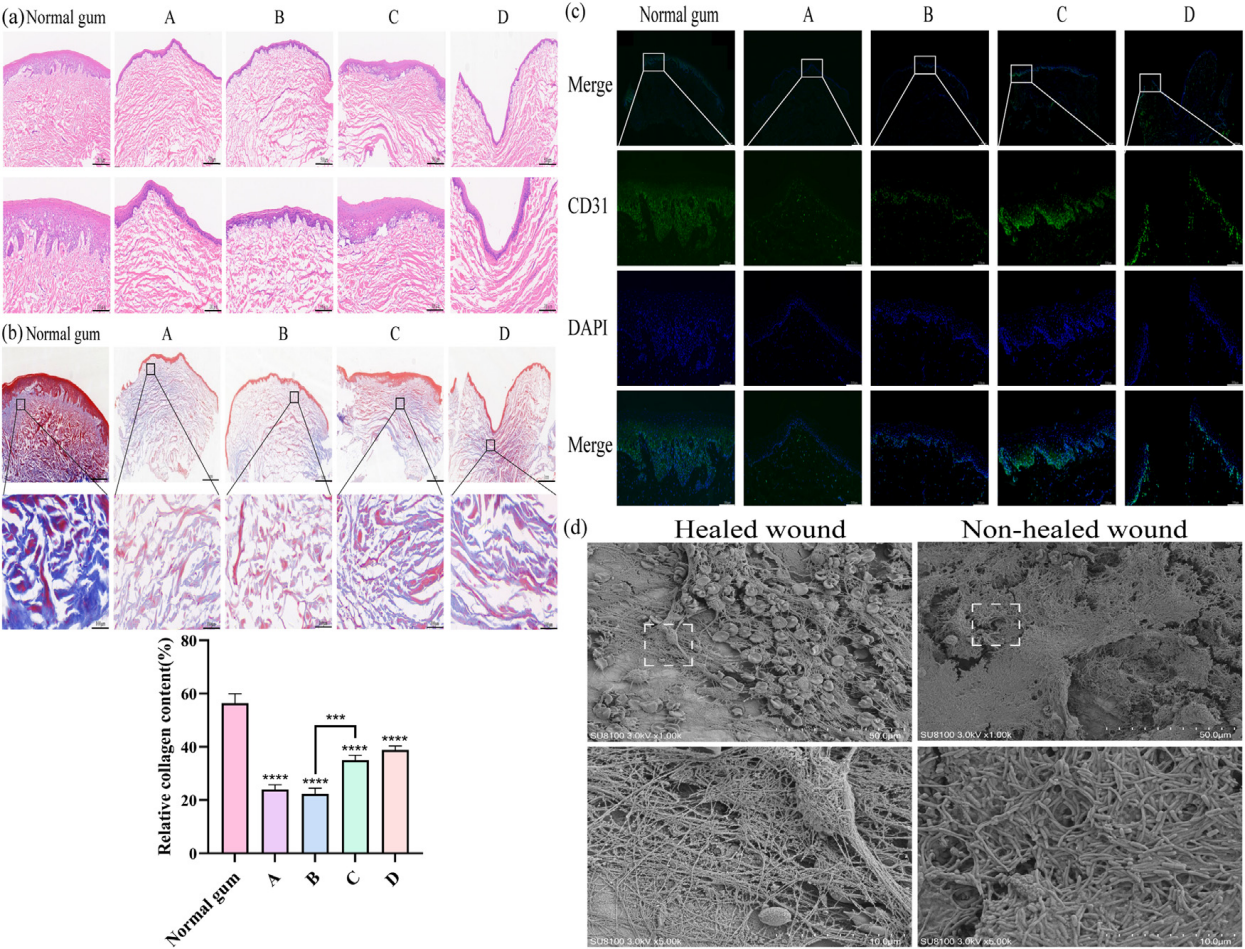
Histological evaluation of the gingiva on the surface of the titanium membrane and SEM images of titanium membranes. (A) HE staining images. Upper row images enlarged 40 ×; lower row images enlarged 100 × . (B) Assessing the collagen fiber content of the gingiva through Masson staining. Upper row enlarged 40 ×; lower row enlarged 100 × . Data are expressed as mean ± SD, and 1-way ANOVA. ^⁎⁎⁎⁎^*P* < .0001, ^⁎⁎⁎^*P* < .001. (C) Assessing the expression level of CD31 in gingiva through immunofluorescence. Upper row enlarged 40 ×; lower 3 rows enlarged 100 × . (D) SEM images of titanium membranes in healed and exposed wounds in group C. The titanium membranes in the upper row have a magnification of 1000. The titanium membranes in the lower row have a magnification of 5000.

From the HE staining images, the thickness of the epithelium in group C was similar to that of normal gums but significantly greater than that in group B (Figure 6A).

Masson staining images showed that the relative collagen fiber content in all experimental groups was lower than that in normal gum (*P* < .0001). However, the relative collagen fiber content in group C was higher than that in group B (*P* < .001; Figure 6B).

Immunofluorescence showed that the expression level of CD31 in group C was higher than that in group B but lower than that in normal gums (Figure 6C).

### SEM analysis

From the SEM images, fibrin and collagen fibers were observed on the surface of the titanium membrane of the healed wound in group C, whereas a large number of bacteria (mainly bacilli) could be seen on the surface of the titanium membrane of the non-healed wound (Figure 6D).

## Discussion

Titanium meshes and membranes are widely used in bone augmentation, especially for large or vertical alveolar bone defects, owing to their superior mechanical support and biocompatibility.^10^ To fully cover the bone graft material and titanium membrane, the mucoperiosteal flaps are usually treated with a periosteal releasing incision to reduce tension. However, using this method, studies have shown that the exposure rate of the titanium mesh can be as high as 51%.^13^ In this experiment, the exposure rates of groups A and B were 50% and 41.67%, respectively, which is consistent with previous reports. Although the literature shows that the standard GBR technique has only 1 type of barrier membrane (resorbable or non-resorbable), some studies have shown that a titanium membrane surface covered with a resorbable collagen membrane (e.g. Bio-gide) could gain more bone augmentation and reduce titanium membrane exposure.^25^, ^26^ Therefore, all groups in this study used double layer membranes (Ti-membrane + Bio-gide). And some studies have shown that the microporous structure and excellent biocompatibility of Bio-oss make it beneficial for the adhesion of bone cells as well as soft tissue cells.^4^ Therefore, compared to group A, a layer of Bio-oss was added between the titanium membrane and Bio-gide to achieve better results in group B,^27^ but there was no statistical difference in the exposure rate between the 2 groups. In Group D, only Ti-membrane and bio-gide were placed to reduce the closing tension of the mucoperiosteal flap, with the aim of verifying the healing ability of beagle dogs' gum on both membrane materials under low tension. And the results confirmed that the gums of beagle dogs could heal well on the surface of Ti-membrane and Bio-gide with an extremely low exposure rate.

There are many reasons for the exposure of titanium mesh; however, in this study, the main reason was that the horizontal periosteal releasing incision affected the blood supply to the mucoperiosteal flap. As evidence, the exposure rates in group C without periosteal releasing incisions were very low. The immunofluorescence results confirmed these findings. Neovascularization of the wound is the main determining factor in wound healing,^28^ and CD31 is a platelet endothelial cell adhesion factor and a commonly used indicator of angiogenesis.^29^ Moreover, CD31 promotes the expression of endoglin, which is a crucial molecule for angiogenesis, vascular development and integrity.^30^ If the periosteal releasing incision leads to decreased expression of CD31, which indicates that this incision has also affected the formation and integrity of blood vessels. In this study, the relative fluorescence intensity of CD31 in group C was higher than that in group B, indicating that the periosteal releasing incision affected the generation of gingival blood vessels. Another piece of evidence was that the relative collagen fiber content and thickness of the epithelium in group C were higher than those in group B, indicating that the gum grew faster in group C.

Although not making a periosteal releasing incision ensures blood supply to the mucoperiosteal flap, as the volume of bone augmentation increases, the tension of the flap inevitably increases. In this experiment, group D only had a titanium membrane placed and the gums could still be closed, while group C accumulated 3 mm of granular bone and the gums could not be closed. Can gums grow on the surface of titanium membranes that are directly exposed to the oral cavity? Clinical evidence shows gums can grow around the healing abutment of titanium, and some researchers have confirmed that gums can heal on the surface of titanium mesh.^21^, ^22^ This is attributed to the excellent biocompatibility of titanium. In this experiment, complete healing of the gums in group C confirmed this point. However, once the surface of the titanium membrane was contaminated with plaque, the gums could not grow on the titanium surface, according to the SEM results in this study. We covered the titanium membrane with a gelatin sponge after surgery to keep it clean. However, the maintenance time was too short. Therefore, there is an urgent need for a material that can cover the surface of the titanium membrane and maintain it for more than 3 weeks to protect it from plaque contamination. Ghanaati et al. covered open healing wounds with sterile latex pieces, which could be maintained until the time for new soft tissue formation.^31^ Stankovic et al. used acellular collagen matrix to close open oral wounds during GBR surgery, achieving good soft tissue healing and morphological appearance.^32^ In addition, the new magnesium membrane with good mechanical strength and absorbency can also serve as an alternative material for Ti-membrane in GBR surgery.^33^ The existence of these alternative materials will promote the achievement of more ideal experimental results in this study.

Although the wound can still heal on the surface of the titanium membrane without a periosteal releasing incision or primary closure of the mucoperiosteal flap, the width of the crack determines the time required for healing. The 3 mm-wide crack in this experiment required approximately 3 weeks of healing time. Although the gum fissures were closed, the degree of keratinization of the gingiva was not high. The epithelial spikes increase with the degree of epithelial cell keratinization.^34^ HE staining showed that the thickness of the epithelium in group C was similar to that of normal gums, but the epithelial spikes were not obvious.

Another advantage of not making a periosteal releasing incision to close the mucoperiosteal flaps is that it widens the keratinized gingiva. Keratinized gingiva plays an important role in resisting mechanical trauma and preventing inflammation around dental implants.^35^ However, implant surgery often reduces keratinized gingiva, particularly in bone augmentation surgery. Some researchers have reported that after undergoing simple and complex bone augmentation surgery, the risk of insufficient keratinized gingiva width around implants is 1.65 times and 2.62 times higher than that of implants without bone augmentation, respectively.^36^ This was mostly due to the overlap of the keratinized gingiva of the buccal and lingual mucoperiosteal flaps to tightly close the wound, similar to groups A and B in this experiment. In group C, there was a certain distance between the keratinized gingiva of the buccal and lingual mucoperiosteal flaps, which provided space for the keratinized gingiva to grow and ultimately widen. As for the long-term impact of not performing periosteal releasing incisions on soft tissue healing, longer-term observation may be required. Ghanaati et al. reported that within 4-8 months after surgery, the newly formed soft tissue in wounds that did not achieve primary closure showed no signs of scar formation or fibrosis.^31^

The control of postoperative oral plaque is crucial to the success of GBR surgery. Jung et al. used 0.2% chlorhexidine to rinse and disinfect the sites of wound cracking and membrane exposure 5-7 weeks after GBR surgery. And the soft tissue at all sites healed before the second phase surgery.^37^ Romas et al. reported that the use of topical antibiotics such as tetracycline hydrochloride could effectively treat peri-implantitis.^38^ Therefore, we also rinsed the postoperative wounds of the beagle dogs with chlorhexidine and metronidazole. The SEM result of group C showed a large number of rod-shaped bacteria on the surface of the titanium mesh exposed to the oral cavity. This might partly be attributed to the lack of cooperation of the beagles, which resulted in wounds not being completely washed clean.

Additionally, in GBR surgery, patients often experience facial swelling and hematoma due to tissue damage or bleeding.^32^ Avoiding periosteal releasing incisions can decrease the incidence of these complications and enhance the patient’s comfort.

In conclusion, a mucoperiosteal flap without a periosteal releasing incision and primary closure can heal on the clean surface of the titanium membrane in vertical bone augmentation, widening the keratinized gingiva. However, the main limitation of this study was the lack of observation of osteogenesis under the titanium membrane because osteogenesis was not completed during the observation time of gum healing. Further studies are required to explore the impact of wounds without primary closure on bone formation. Meanwhile, alternative materials used to cover open healing wounds also deserve further research.

## Declaration of competing interest

The authors declare that they have no known competing financial interests or personal relationships that could have appeared to influence the work reported in this paper.

## Appendix Supplementary materials

Image, application 1

## Footnotes

Supplementary material associated with this article can be found, in the online version, at doi:10.1016/j.identj.2025.04.004.

## References

[1] Chiapasco M., Casentini P. (2018). Horizontal bone-augmentation procedures in implant dentistry: prosthetically guided regeneration. Periodontol 2000.

[2] Blanco J., Alonso A., Sanz M. (2005). Long-term results and survival rate of implants treated with guided bone regeneration: a 5-year case series prospective study. Clin Oral Implants Res.

[3] Buser D., Dula K., Belser U.C., Hirt H.P., Berthold H. (1995). Localized ridge augmentation using guided bone regeneration. II. Surgical procedure in the mandible. Int J Periodontics Restorative Dent.

[4] Zhao R., Yang R., Cooper P.R., Khurshid Z., Shavandi A., Ratnayake J. (2021). Bone grafts and substitutes in dentistry: a review of current trends and developments. Molecules.

[5] Benic G.I., Hämmerle CH. (2014). Horizontal bone augmentation by means of guided bone regeneration. Periodontol 2000.

[6] Owens K.W., Yukna RA. (2001). Collagen membrane resorption in dogs: a comparative study. Implant Dent.

[7] Xie Y., Li S., Zhang T., Wang C., Cai X. (2020). Titanium mesh for bone augmentation in oral implantology: current application and progress. Int J Oral Sci.

[8] Lin W.S., Starr T.L., Harris B.T., Zandinejad A., Morton D. (2013). Additive manufacturing technology (direct metal laser sintering) as a novel approach to fabricate functionally graded titanium implants: preliminary investigation of fabrication parameters. Int J Oral Maxillofac Implants.

[9] Schrank E.S., Hitch L., Wallace K., Moore R., Stanhope S.J. (2013). Assessment of a virtual functional prototyping process for the rapid manufacture of passive-dynamic ankle-foot orthoses. J Biomech Eng.

[10] Jung G.U., Jeon J.Y., Hwang K.G., Park CJ. (2014). Preliminary evaluation of a three-dimensional, customized, and preformed titanium mesh in peri-implant alveolar bone regeneration. J Korean Assoc Oral Maxillofac Surg.

[11] Sidambe AT. (2014). Biocompatibility of Advanced Manufactured Titanium Implants-A Review. Materials (Basel).

[12] Cordeiro J.M., Barão VAR. (2017). Is there scientific evidence favoring the substitution of commercially pure titanium with titanium alloys for the manufacture of dental implants?. Mater Sci Eng C Mater Biol Appl.

[13] Zhou L., Su Y., Wang J., Wang X., Liu Q., Wang J. (2022). Effect of exposure rates with customized versus conventional titanium mesh on guided bone regeneration: systematic review and meta-analysis. J Oral Implantol.

[14] Rider P., Kačarević Ž P, Elad A. (2022). Biodegradable magnesium barrier membrane used for guided bone regeneration in dental surgery. Bioact Mater.

[15] Cunha G., Carvalho P.H.A., Quirino L.C. (2022). Titanium mesh exposure after bone grafting: treatment approaches-a systematic review. Craniomaxillofac Trauma Reconstr.

[16] Gutta R., Baker R.A., Bartolucci A.A., Louis PJ. (2009). Barrier membranes used for ridge augmentation: is there an optimal pore size?. J Oral Maxillofac Surg.

[17] Ogata Y., Griffin T.J., Ko A.C., Hur Y. (2013). Comparison of double-flap incision to periosteal releasing incision for flap advancement: a prospective clinical trial. Int J Oral Maxillofac Implants.

[18] Xiong Y., Lin Z., Bu P. (2023). A whole-course-repair system based on neurogenesis-angiogenesis crosstalk and macrophage reprogramming promotes diabetic wound healing. Adv Mater.

[19] Yang J., Chen Z., Pan D., Li H., Shen J. (2020). Umbilical cord-derived mesenchymal stem cell-derived exosomes combined Pluronic F127 hydrogel promote chronic diabetic wound healing and complete skin regeneration. Int J Nanomedicine.

[20] Romanos GE. (2010). Periosteal releasing incision for successful coverage of augmented sites. A technical note. J Oral Implantol.

[21] Ku J.K., Leem DH. (2019). Vestibuloplasty covering titanium mesh with grafted free gingiva on anterior mandible: technical report and rationale. J Korean Assoc Oral Maxillofac Surg.

[22] Ku J.K., Leem DH. (2020). Retrospective case series analysis of vestibuloplasty with free gingival graft and titanium mesh around dental implant. J Korean Assoc Oral Maxillofac Surg.

[23] Kilkenny C., Browne W., Cuthill I.C., Emerson M., Altman DG. (2010). Animal research: reporting in vivo experiments: the ARRIVE guidelines. J Gene Med.

[24] Ko K.A., Lee J.S., Kim J.H., Park J.M., Gruber R., Thoma DS. (2020). Changes in mucogingival junction after an apically positioned flap with collagen matrix at sites with or without previous guided bone regeneration: a prospective comparative cohort study. Clin Oral Implants Res.

[25] Cucchi A., Vignudelli E., Napolitano A., Marchetti C., Corinaldesi G. (2017). Evaluation of complication rates and vertical bone gain after guided bone regeneration with non-resorbable membranes versus titanium meshes and resorbable membranes. A randomized clinical trial. Clin Implant Dent Relat Res.

[26] Shin S.I., Herr Y., Kwon Y.H., Chung JH. (2013). Effect of a collagen membrane combined with a porous titanium membrane on exophytic new bone formation in a rabbit calvarial model. J Periodontol.

[27] Proussaefs P., Lozada J. (2006). Use of titanium mesh for staged localized alveolar ridge augmentation: clinical and histologic-histomorphometric evaluation. J Oral Implantol.

[28] Martin P. (1997). Wound healing–aiming for perfect skin regeneration. Science.

[29] Figueiredo C.C., Pereira N.B., Pereira L.X. (2019). Double immunofluorescence labeling for CD31 and CD105 as a marker for polyether polyurethane-induced angiogenesis in mice. Histol Histopathol.

[30] Park S., Sorenson C.M., Sheibani N. (2015). PECAM-1 isoforms, eNOS and endoglin axis in regulation of angiogenesis. Clin Sci (Lond).

[31] Ghanaati S., Al-Maawi S., Conrad T., Lorenz J., Rössler R., Sader R. (2019). Biomaterial-based bone regeneration and soft tissue management of the individualized 3D-titanium mesh: An alternative concept to autologous transplantation and flap mobilization. J Craniomaxillofac Surg.

[32] Stankovic D., Labudovic-Borovic M., Radosavljevic R., Marinkovic M., Isenovic ER. (2018). Use of acellular collagen matrix for the closure of the open oral wound in bone regeneration. J Stomatol Oral Maxillofac Surg.

[33] Rider P., Kačarević Ž P, Elad A. (2022). analysis of a pure magnesium membrane degradation process and its functionality when used in a guided bone regeneration model in beagle dogs. Materials (Basel).

[34] Shan X., Han D., Ge Y., Zhang H., Lu R. (2022). Clinical outcomes of keratinized mucosa augmentation in jaws reconstructed with fibula or iliac bone flaps. Int J Oral Maxillofac Surg.

[35] Souza A.B., Tormena M., Matarazzo F., Araújo MG. (2016). The influence of peri-implant keratinized mucosa on brushing discomfort and peri-implant tissue health. Clin Oral Implants Res.

[36] Wang Q., Tang Z., Han J., Meng H. (2020). The width of keratinized mucosa around dental implants and its influencing factors. Clin Implant Dent Relat Res.

[37] Jung R.E., Hälg G.A., Thoma D.S., Hämmerle CH. (2009). A randomized, controlled clinical trial to evaluate a new membrane for guided bone regeneration around dental implants. Clin Oral Implants Res.

[38] Ramos U.D., Suaid F., Wikesjö U.M.E. (2018). Microbiologic effect of 2 topical anti-infective treatments on ligature-induced peri-implantitis: a pilot study in dogs. J Periodontol.

