# Supplementary material for: Mucoperiosteal Flap Healing During Vertical Bone Augmentation using Titanium Mesh: A Study in Beagle Dogs
Source: Int Dent J. 2025 May 9;75(4):100823. doi: 10.1016/j.identj.2025.04.004 (PMC12139418; doi:10.1016/j.identj.2025.04.004)
Supplement: Supplementary file 1 [file mmc1.docx]

Supplementary table 1 Allocation of groups based on different tooth sites in beagle dogs.

| Dogs’ number | 1 | | 2 | | | 3 | | | 4 | | | 5 | | 6 | |  |
| --- | --- | --- | --- | --- | --- | --- | --- | --- | --- | --- | --- | --- | --- | --- | --- | --- |
|  | Left | Right | | Left | Right | | Left | Right | | Left | Right | Left | Right | Left | Right | |
| P2 | A | B | | C | D | | A | C | | B | D | D | A | B | C | |
| P3 | C | D | | A | B | | C | D | | A | C | B | B | A | D | |
| P4 | D | A | | B | C | | B | A | | C | A | C | D | D | B | |
| M1 | B | C | | D | A | | D | B | | D | B | A | C | C | A | |

Annotation:

1, 2, 3, 4, 5, 6: the number/ID of beagle dogs.

Left, Right: The left or right mandible of beagle dogs.

P2: second premolar.

P3: third premolar.

P4: fourth premolar.

M1: first molar.
